# Attenuation of doxorubicin-induced cardiotoxicity by *cryptotanshinone* detected through association analysis of transcriptomic profiling and KEGG pathway

**DOI:** 10.18632/aging.103228

**Published:** 2020-05-26

**Authors:** Le Li, Bin Wu, Qiangqiang Zhao, Jian Li, Yunfeng Han, Xiaohang Fan, Junli Dong, Pengcheng Li

**Affiliations:** 1 Department of Cardiology, The Third Xiangya Hospital, Central South University, Changsha, China; 2 Laboratory of Platelet and Endothelium Biology, Department of Transfusion Medicine, Wuhan Hospital of Traditional Chinese and Western Medicine (Wuhan No.1 Hospital), Tongji Medical College, Huazhong University of Science and Technology, Wuhan, China; 3 Department of Blood Transfusion, The Third Xiangya Hospital, Central South University, Changsha, China; 4 Department of Hematology, Qinghai Provincial People’s Hospital, Xi’ning, China; 5 Department of Nuclear Medicine, Tongji Hospital, Tongji Medical College, Huazhong University of Science and Technology, Wuhan, China; 6 Department of Pathophysiology, Scholl of Basic Medicine, Tongji Medical College, Huazhong University of Science and Technology, Wuhan, China; 7 Laboratory of Clinical Pharmacogenetics, Department of Pharmacy, Wuhan Hospital of Traditional Chinese and Western Medicine, Tongji Medical College, Huazhong University of Science and Technology, Wuhan, China

**Keywords:** doxorubicin, cryptotanshinone, cardiotoxicity, reactive oxygen species, transcriptomic profiling

## Abstract

Objective: The cardiotoxicity of doxorubicin (DOX) reduces the quality of life and prognosis of cancer patients, and therefore its clinical application has been largely restricted. This study aimed to assess the effects of cryptotanshione (CPT) on DOX-induced rat cardiac insufficiency.

Results: CPT treatment significantly suppressed apoptosis *in vitro*. The oral administration of CPT significantly improved cardiac function in the rat model, reduced collagen production and suppressed apoptosis and the production of reactive oxygen species in the heart tissue. Transcriptomic profiling and its relevant bioinformatics analysis showed that CPT suppressed doxorubicin-induced cardiotoxicity by inhibiting p53 signaling pathway.

Conclusion: Transcriptomic profiling and bioinformatics analysis can be used to evaluate the cardio-protective effect of CPT through inactivating p53 signaling pathway in the doxorubicin-mediated myocardial damage model.

Methods: F-actin staining and flow cytometry were used to assess the effects of CPT on cardiomyocytes. *In vivo*, echocardiography and hemodynamic evaluation were used to assess the effects of CPT on the cardiac dysfunction in rats. Furthermore, transcriptomic profiling and bioinformatics analysis, as well as western blot analysis, were used to determine that CPT induced changes in the signaling pathways in the model.

## INTRODUCTION

The anthracycline antibiotic doxorubicin (DOX) is a common and effective antitumor drug with a wide range of applications [1]. However, chemotherapeutic drugs not only effectively kill tumor cells but also damage the normal tissues and cells due to their lack of selectivity, leading to indigestion, fatigue, massive hair loss and cardiotoxicity [2]. These side effects are common among cancer patients using DOX for chemotherapy and severely reduce the quality of life and prognosis of patients [3]. The DOX-induced cardiotoxicity is often manifested by cardiomyopathy, left ventricular (LV) dysfunction, arrhythmia and congestive heart failure [4], among which cardiomyopathy is the most severe side effect [5, 6]. Previous studies have shown that the inhibition of nucleotide and protein synthesis [7], changes in the transport of calcium ions in cardiomyocytes [8], and expressions of some DOX-sensitive transcription factors [9] are associated with DOX-induced cardiotoxicity. However, the drastic increase in the levels of reactive oxygen species (ROS) and a significant reduction in antioxidant levels are the most important factors for DOX-induced myocardial lesions [10]. Cadete et al. [11] found that DOX promoted the formation of mitochondrial microparticles in cardiomyocytes and selectively transported these damaged mitochondrial materials to the lysosomes in the DOX-induced mouse myocardial damage model, ultimately leading to a severe decline in the mitochondrial function of cardiomyocytes and triggering an imbalance in myocardial energy metabolism. When the energy supply by the cardiomyocytes is not sufficient for cardiac contraction, the impaired cardiac energy metabolism eventually leads to cardiac insufficiency and heart failure if the production of ROS-mediated pro-oxidants cannot be timely suppressed by antioxidant treatment [12].

**Figure 1:**
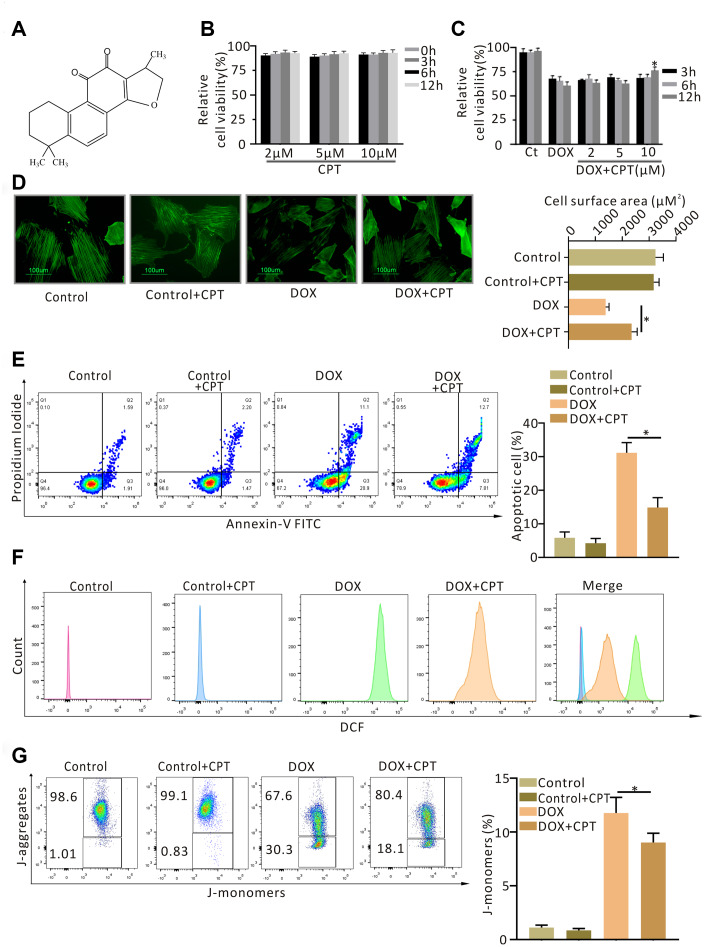
**Cryptotanshinone (CPT) protected H9c2 cardiomyocytes against doxorubicin-induced damage.** Chemical structure of CPT (**A**). Cytotoxicity of CPT *in vitro* (**B**). Effects of CPT on the viability of H9c2 cardiomyocytes induced by various concentrations of doxorubicin at the indicated time-points (**C**). Cell-size measurement of H9c2 cardiomyocytes (green-F-actin staining) induced by doxorubicin in the presence or absence of CPT treatment (**D**). The number of apoptotic H9c2 cells determined using Annexin V/ Propidium Iodide (PI) staining (**E**). Assessment of reactive oxygen species (ROS) by flow cytometry analysis after treatment with doxorubicin in the presence or absence of CPT (**F**). The JC-1 monomers and aggregates of H9c2 cells stimulated by doxorubicin with/without CPT detected by flow cytometry (**G**). Values are mean ± standard error of the mean; all experiments were performed in three replicates. ^*^Significant difference (*P*<0.05), DOX + CPT vs DOX.

Danshen (*Salvia miltiorrhiza Bunge*) is a common medicinal ingredient. It has been used as a medicated diet in northeastern Asia for thousands of years [13]. More than 90 natural compounds extracted from danshen have been identified to date. Some components have been shown to have pharmacological activities, including anti-inflammatory, antitumor, antidiabetic and neuroprotective effects. A recent study showed that cryptotanshinone (CPT) (Figure 1A), one of the extracts from danshen, inhibits atherosclerotic plaque formation in ApoE^-/-^ mice; and the underlying mechanism is through suppressing the production of ROS and reducing the expression of extracellular matrix proteins [14]. Another study also confirmed that CPT inhibited the proliferation of human melanoma cell lines *in vitro* by upregulating the activities of pro-apoptotic proteins, including Bcl-2 associated x (Bax) and cleaved caspase-3 [15]. An earlier study found that CPT alleviated mitochondrial apoptosis of cardiomyocytes through decreasing the Bax expression and reducing caspase-3 activation in the *in vitro* H9c2 cardiomyocyte injury model induced by chronic hypoxia, thereby protecting cardiomyocytes [16]. Importantly, Zhang et al. [17] found that CPT can significantly increase antioxidant levels in rat hearts. However, Zhang et al. [17] only found that CPT increased the antioxidant capacity in the heart of the DOX-induced cardiotoxicity rat model but they did not show whether CPT had any effect on the cardiac function of rats. Multiple interconnected signaling pathways are involved in DOX-mediated cardiomyocyte injury. However, no study reported the use of transcriptomic profiling and related Kyoto Encyclopedia of Genes and Genomes (KEGG) analysis to determine the crucial signaling pathways through which CPT attenuated DOX-induced cardiac damage.

Therefore, this study aimed to assess the effects of CPT on DOX-induced cardiac insufficiency in rats. Importantly, the transcriptomic profiling analysis was used to investigate the changes mediated by CPT in DOX-treated cardiomyocytes and, through these changes, determine which signaling pathways were involved in CPT-mediated protection of hearts from DOX-induced cardiotoxicity.

## RESULTS

### Effects of CPT on cardiomyocyte viability

CPT at different concentrations did not decrease the viability of myocardial H9c2 cells at all the time points examined (Figure 1B). DOX treatment significantly reduced H9c2 cell viability, which was ameliorated by 10μM CPT treatment for 12 h (*P*<0.05, Figure 1C).

### Effects of CPT on the surface area of cardiomyocytes

H9c2 cells were stained with F-actin, and the changes in the cell surface area were examined to determine the effects of CPT on DOX-induced changes in H9c2 cells *in vitro*. No significant difference was found in the surface area of cardiomyocytes between the control (Ct) group and the Ct + CPT group (Figure 1D). Treating H9c2 cells with DOX for 12h significantly reduced the surface area of cardiomyocytes (*P*<0.05, Figure 1D). However, the cells treated with 10μM CPT for 12h had a significantly increased cardiomyocyte surface area compared with the cells treated with DOX alone (*P*<0.05, Figure 1D).

### Effects of CPT on DOX-induced H9c2 apoptosis, ROS production, and decline in mitochondrial membrane potential

Apoptosis, ROS production and mitochondrial membrane potential (MMP) in H9c2 cardiomyocytes were assessed using flow cytometry. Treating H9c2 cells with DOX for 12 h significantly increased ROS production, reduced MMP, and induced apoptosis (*P*<0.05; Figure 1E–1G). Further, 10 μM CPT significantly suppressed DOX-induced H9c2 apoptosis, ROS production, and MMP reduction. Moreover, CPT had no obvious effect on the apoptosis rate, level of ROS production, and MMP in H9c2 cardiomyocytes (Figure 1E–1G).

### CPT alleviated DOX-induced cardiac dysfunction in rats

**Figure 2:**
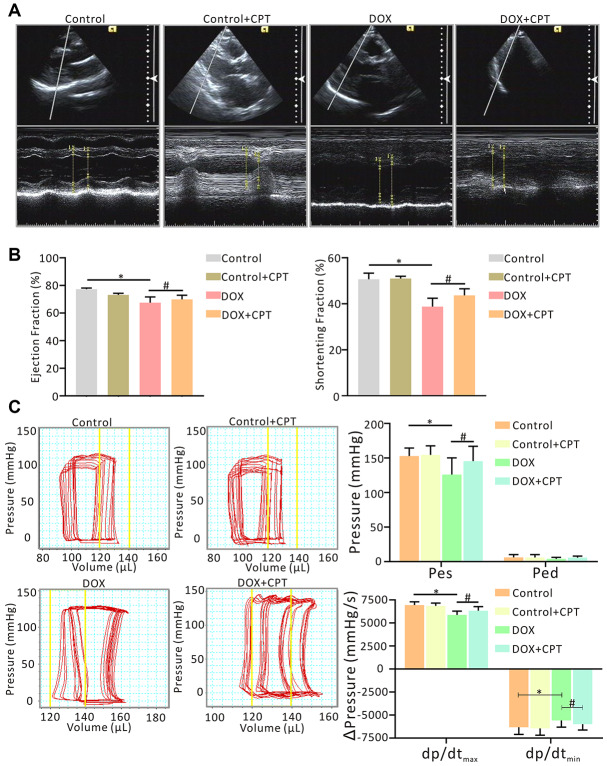
**Oral administration of cryptotanshinone (CPT) ameliorated cardiac dysfunction in doxorubicin-induced injury.** Evaluation of cardiac function by echocardiography with/without CPT treatment, indicated by ejection fraction (EF, %) of LV and fractional shortening (FS, %) of LV, in the doxorubicin-induced rat model (**A, B**). Measurement of invasive hemodynamic examinations for analyzing both LV pressure and volume (pressure-volume loop) simultaneously, also in the rats model, indicated by end-systolic pressure (Pes), end-diastolic pressure (Ped), maximal rates of the increase in ventricular pressure (dp/dt_max_) and maximal rates of decline in ventricular pressure (dp/dt_min_) (**C**). ^*^P<0.05 vs control, ^#^*P*<0.05 vs DOX.

In this study, echocardiography and hemodynamic examination were conducted to evaluate cardiac function in rats. The cardiac function of the Wistar rats significantly reduced after six injections of DOX within 2 weeks. The ejection fraction (EF) (%) and shortening fraction (FS) (%) of the rats in the DOX group significantly reduced (*P*<0.05; Figure 2A and 2B). Moreover, the hemodynamic examination also showed that DOX induced a left shift of the pressure-volume (PV) loop. Importantly, the EF (%) and FS (%) significantly increased in rats orally administered with CPT and DOX injection compared with those treated with DOX alone (*P*<0.05; Figure 2A and 2B). Meanwhile, CPT also induced a significant right shift of the PV loop along the horizontal axis (Figure 2C). These results indicated that CPT treatment alleviated DOX-induced rat cardiac insufficiency. Further, the rat cardiac function was neither upregulated nor downregulated by the oral administration of CPT in the Ct + CPT group compared with the Ct group (Figure 2).

### Effects of CPT on DOX-induced rat cardiac histology

**Figure 3:**
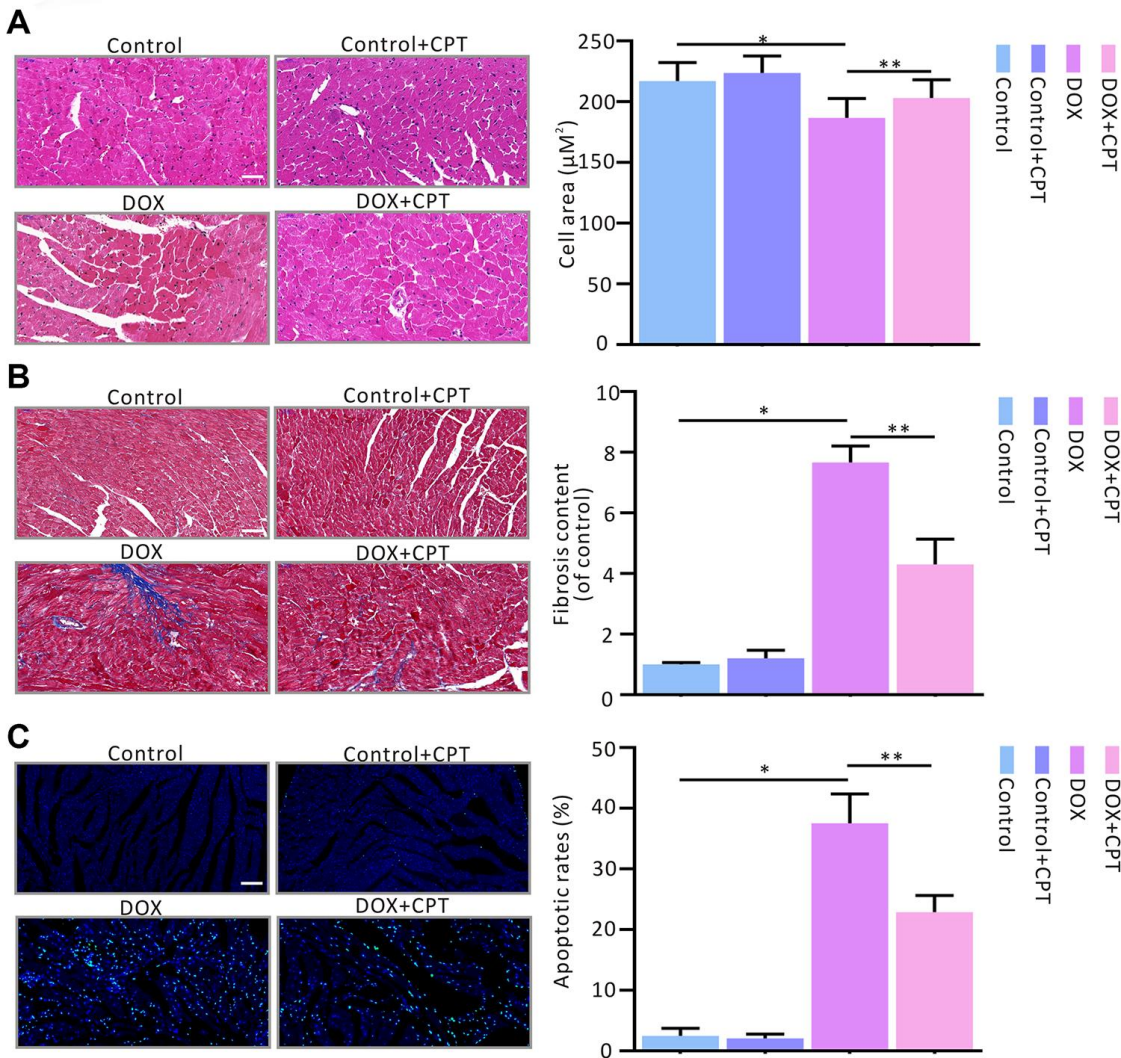
**Cryptotanshinone (CPT) gavage attenuated myocardial fibrosis and apoptosis in rats treated with doxorubicin.** Hematoxylin and eosin staining for paraffin section (**A**). Masson’s trichrome staining for fibrosis (**B**). TUNEL assay for apoptotic cardiac cells (**C**). Values are expressed as mean ± standard error of the mean. ^*^P<0.05 vs control, ^#^*P*<0.05 vs DOX. Scale bar: 50 μm.

The cardiac histology was assessed using hematoxylin-eosin (HE), Masson’s trichrome stain (MTS) and terminal deoxynucleotidyl transferase-mediated dUTP nick-end labeling (TUNEL) to evaluate the effects of CPT on DOX-induced changes in rat cardiac histological morphology. At the end of the observation after the injection of Wistar rats with DOX, cavities were found in the nucleus; also, the cardiomyocytes were loosely aligned, and the surface areas of the cardiomyocytes significantly reduced (*P*<0.05; Figure 3A). However, following 6 weeks of CPT treatment, the number of hollow nuclei reduced, the cardiomyocytes were more tightly aligned, and the surface areas of the cardiomyocytes significantly increased (*P*<0.05; Figure 3A). DOX induced significant collagen deposition in the hearts of rats, and the level of cardiac collagen significantly increased compared with that in control Wistar rats (*P*<0.05; Figure 3B). Further, TUNEL assay showed that CPT significantly reduced the DOX- induced cardiomyocyte apoptosis rate in the hearts of rats (*P*<0.05; Figure 3C). Moreover, consistent with the previous findings, CPT treatment did not cause collagen deposition and apoptosis in the hearts of rats compared with the control group (Figure 3).

### CPT reversed DOX-induced MMP reduction and suppressed ROS production in rat cardiomyocytes

**Figure 4:**
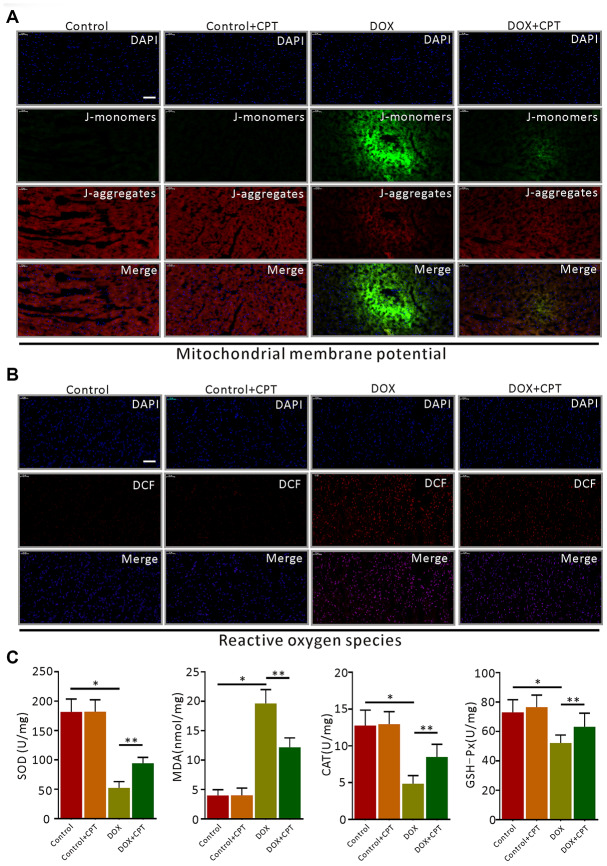
**Cryptotanshinone (CPT) alleviated myocardial mitochondrial membrane potential (MMP) decline and oxidative stress in rats treated with doxorubicin.** JC-1 fluorescent mitochondrial depolarization of LV cardiomyocytes for evaluating MMP (**A**). Detection of intracellular ROS by observing the fluorescence intensity of DCF in the LV of the rats (**B**). The levels of SOD, MDA, CAT, and GSH-Px in LV tissues of the rats induced by doxorubicin (**C**). Values are expressed as mean ± standard error of the mean. ^*^P<0.05 vs control, ^#^*P*<0.05 vs DOX. CAT, Catalase; GSH-Px, glutathinone peroxidase; MDA, malonydialdehyde; SOD, superoxide dismutase. Scale bar: 50 μm.

DOX significantly downregulated MMP levels (Figure 4A) and drastically increased ROS levels in rat cardiomyocytes (Figure 4B). After 6-week CPT treatment, the DOX-induced reduction in cardiac MMP levels in rats was significantly attenuated, and ROS levels were suppressed (Figure 4A and 4B). However, its intervention did not upregulate the levels of MMP and ROS in cardiomyocytes of the Ct + CPT group compared with of the Ct group (Figure 4A and 4B). Also, the levels of antioxidants and pro-oxidants in the cardiomyocytes of rats were measured, revealing that CPT significantly elevated the superoxide dismutase (SOD), catalase (CAT) and glutathione peroxidase (GSH-Px) levels but reduced the malondialdehyde (MDA) level (*P*<0.05; Figure 4C). Consistent with the aforementioned results, no significant difference was found in the levels of antioxidants and oxidants between the Ct and the Ct + CPT groups (Figure 4C). These results indicated that CPT suppressed DOX-induced ROS production and reversed DOX-induced MMP reduction. Meanwhile, CPT improved the antioxidant capacity of the hearts of the rats.

### Transcriptomic profiling and KEGG pathway analysis

**Figure 5:**
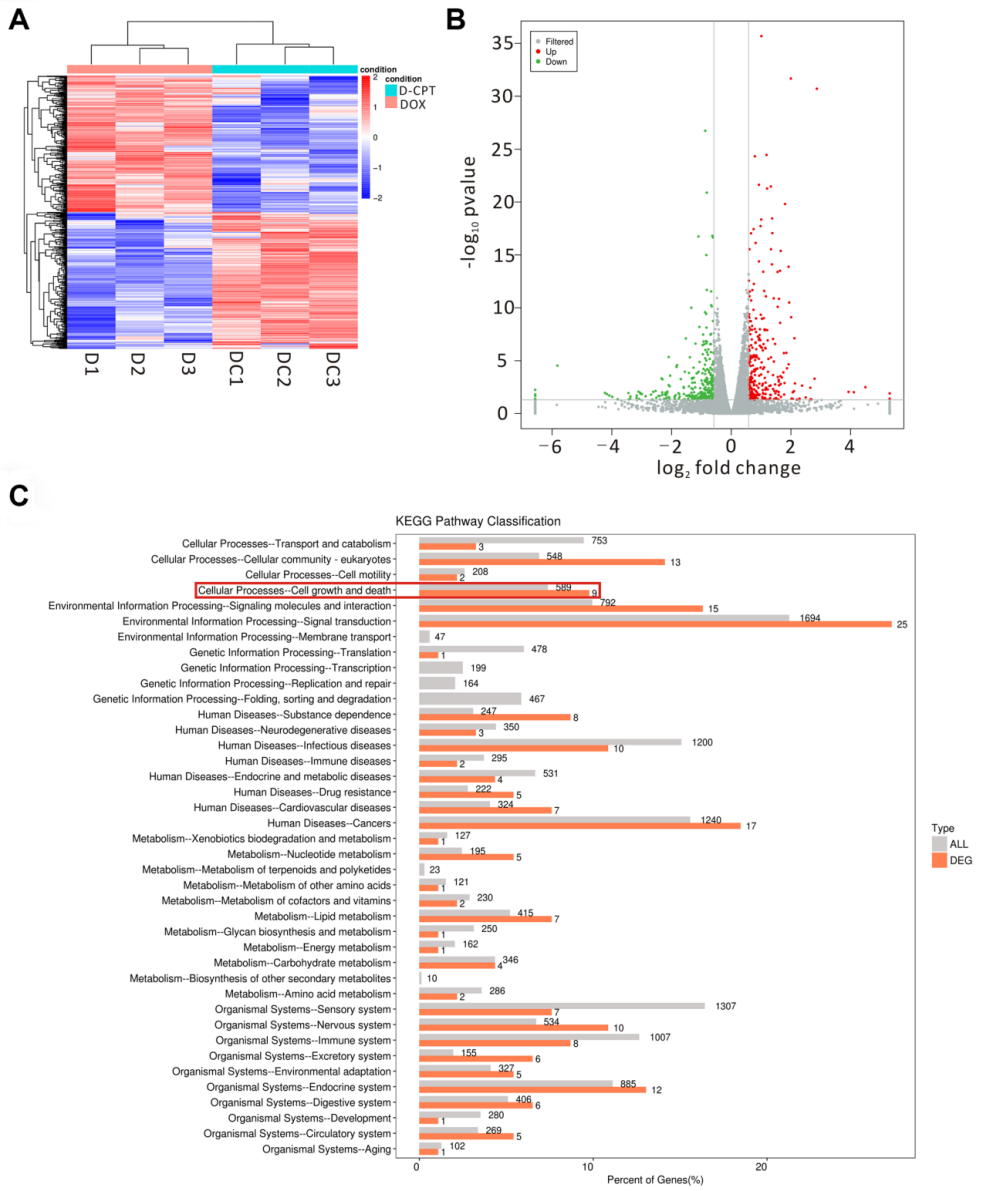
**Effects of the different regulated genes and pathways of cryptotanshinone (CPT) on the heart tissues of the DOX-treated rats using transcriptomic profiling.** Changed genes in the heart tissues of the two groups displayed by a heat map (D1-3 = DOX group, DC1-3 = DOX + CPT group) (**A**). The volcano map exhibits the differentially regulated genes by comparison of the two groups (DOX and DOX + CPT groups); the gray scatter denotes the non-significantly differentially expressed genes; both red and green scatters denote the significantly differentially expressed genes; the x-axis represents log_2_ fold change, and the y-axis represents the –log_10_ *P* value (**B**). KEGG pathway classification; the horizontal axis represents the ratio (%) of the total number of genes (differentially expressed genes) annotated with each level 2 metabolic pathway and the genes (differentiated genes) annotated to the KEGG pathway, and the vertical axis represents the level 2 pathway term; the number on the right side of the column represents the annotation of the number of differentially expressed genes in the level 2 pathway term (**C**).

**Figure 6:**
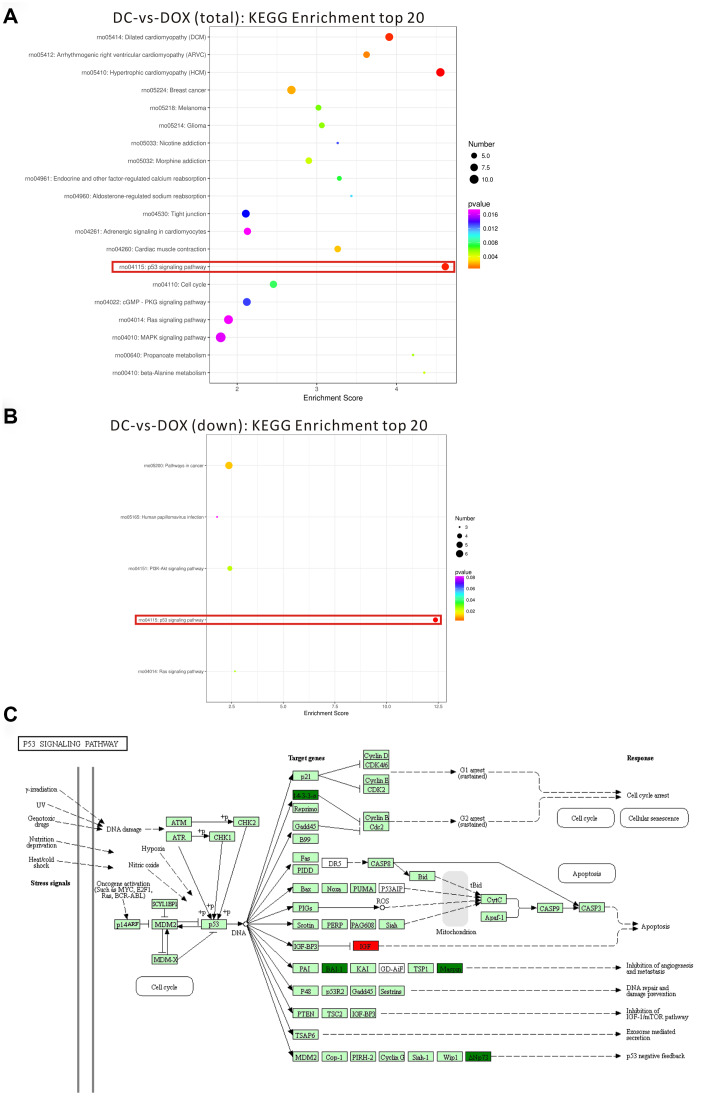
**Top 20 of pathway enrichment statistics based on the differentially expressed gene in the rat hearts after cryptotanshinone (CPT) treatment.** Scatter plot of KEGG pathway enrichment statistics (the total number of differentially expressed genes) (**A**). Scatter plot of KEGG pathway enrichment statistics (the number of differentially expressed genes, which were downregulated (**B**). p53 signaling pathway in a KEGG map (**C**). Enrichment score is the ratio of the number of differentially expressed genes to the number of all genes in this pathway term.

The changes in cardiac gene expression was examined using transcriptomic profiling to investigate the mechanism underlying the cardioprotective function of CPT in the DOX-induced cardiotoxicity rat model (Figure 5A and 5B). The results indicated that 589 differentially expressed genes were closely involved in cell growth and death (comparison between the DOX + CPT and DOX groups, with *P*<0.05 indicating a significant difference in gene expression levels between the two groups, Figure 5C). Considering the cardiotoxicity of DOX and that KEGG analysis of the differentially expressed genes showed a difference in the p53 signaling pathway, the following experiments focused on the effects of CPT and DOX on the p53 signaling pathway that is involved in the apoptosis of cardiomyocytes of rats (Figure 6A–6C).

### Effects of CPT on the expressions of signaling proteins of p53 pathway

**Figure 7:**
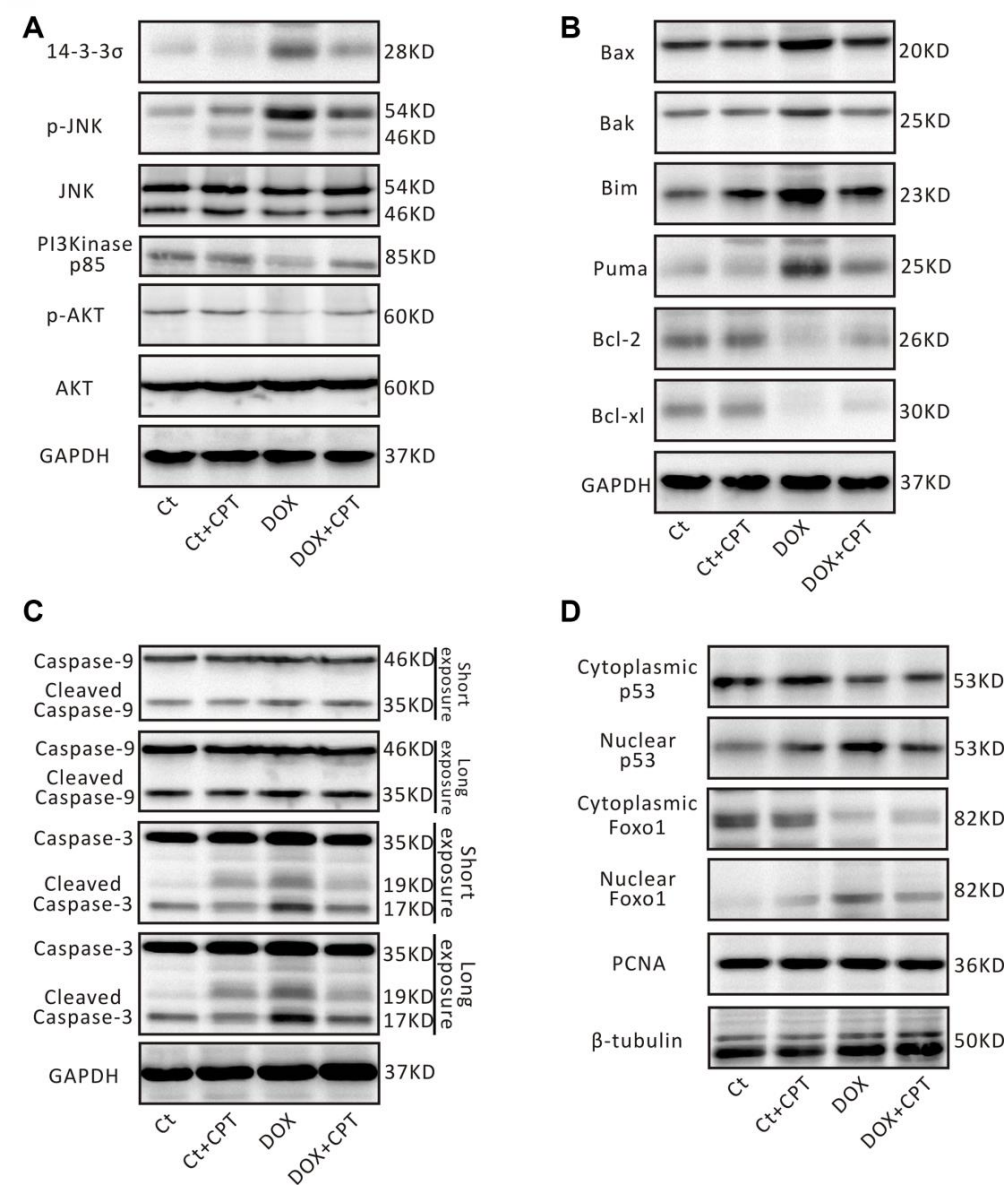
**Cryptotanshinone (CPT) treatment adjusted p53 signaling pathway in the rat heart induced by doxorubicin.** Representative blots of 14-3-3σ, p-JNK, JNK, PI3K p85, p-AKT, AKT, and GAPDH (**A**). Representative blots of Bax, Bak, Bim, PUMA, Bcl-2, Bcl-xl, and GAPDH (**B**). Representative blots of pro-caspase-3/cleaved caspase-3, pro-caspase-9/cleaved caspase-9, and GAPDH (C). Representative blots of Foxo1, p53, PCNA, and β-tubulin (**D**). GAPDH, β-tubulin and PCNA were used as internal reference.

**Figure 8:**
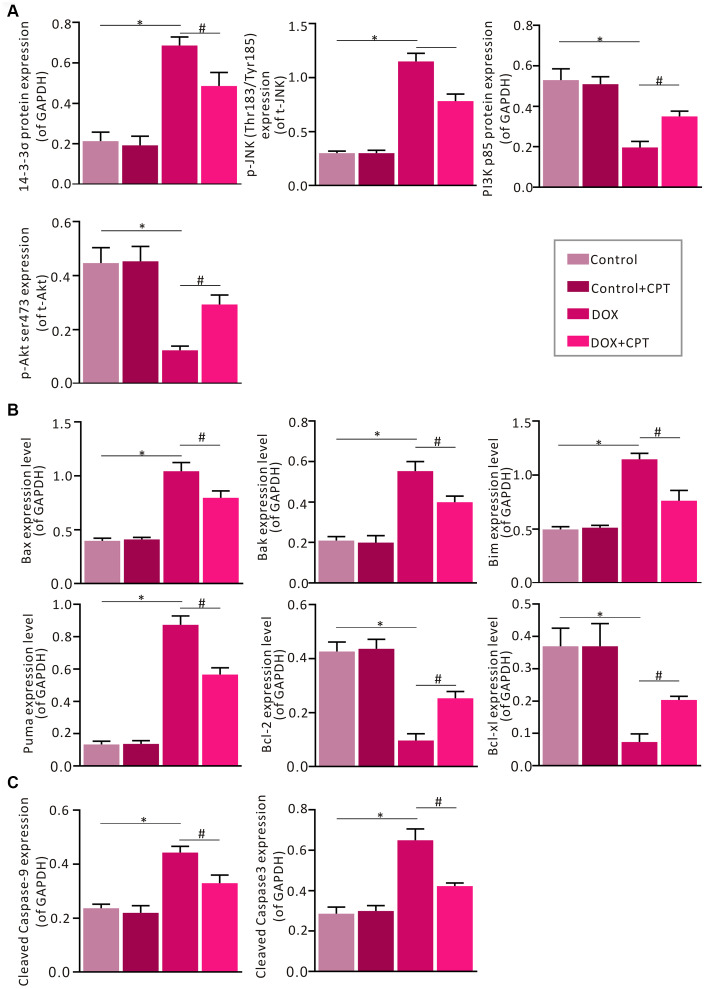
**Statistical analysis of the relative protein expression of 14-3-3σ, PI3K p85, p-JNK, p-AKT, cleaved caspase-9, cleaved caspase-3, Bax, Bak, Bim, PUMA, Bcl-xl, and Bcl-2.** Semi-qualification of 14-3-3σ, PI3K p85, p-JNK and p-AKT (**A**). Semi-qualification of Bax, Bak, Bim, PUMA, Bcl-xl, and Bcl-2 (**B**). Semi-qualification of cleaved caspase-9 and cleaved caspase-3 (**C**). Both GAPDH and PCNA were used as internal reference. ^*^P<0.05 vs control, ^#^*P*<0.05 vs DOX.

The effects of CPT on the expression of some critical signaling proteins in this pathway were examined by Western blot analysis to confirm the changes in the expressions of proteins related to the p53 signaling pathway identified in using transcriptome sequencing and bioinformatics analysis. After the injection of DOX into Wistar rats, the expression levels of myocardial 14-3-3σ and p-c-Jun N-terminal kinase (JNK) were significantly elevated in the rats of the DOX group (*P*<0.05; Figures 7A and 8A). Meanwhile, the expression levels of PI3 kinase p85 and p-AKT were all significantly downregulated (*P*<0.05; Figures 6A and 7A). However, after treatment of DOX-injected rats with CPT for 6 weeks, the expression levels of PI3 kinase p85 and p-AKT were all significantly upregulated compared with those in the DOX group (*P*<0.05; Figures 6A and 7A). CPT further suppressed the DOX-induced 14-3-3σ expression and p-JNK phosphorylation (*P*<0.05; Figures 6A and 7A). Moreover, CPT intervention alone did not affect the expression level and the phosphorylation level of the aforementioned proteins, including 14-3-3σ, p85, p-JNK and p-AKT, in the hearts of Wistar rats (*P*<0.05; Figures 6A and 7A).

Additionally, the DOX-induced downregulation of protein expression of B-cell lymphoma-2 (Bcl-2) and B-cell lymphoma-extra large (Bcl-xl) in rat cardiomyocytes was attenuated by CPT treatment (*P*<0.05; Figures 7B and 8B). Meanwhile, the expressions of cardiac Bcl-2 associated x (Bax), Bcl-2-antagonist/killer (Bak), Bcl-2 interacting mediator of cell death (Bim), and P53 up-regulated modulator of apoptosis (PUMA) in the rats of the DOX and DOX + CPT groups. CPT significantly suppressed the expressions of these proteins (*P*<0.05; Figures 7B and 8B). Cleaved caspase-9 and cleaved caspase-3, related to mitochondria apoptosis, were significantly upregulated. However, CPT significantly suppressed the cleavage of caspase-9 and caspase-3 (*P*<0.05; Figures 7C and 8C).

The nuclear translocation of p53 and Foxo1 in the myocardium was examined. The findings revealed that cytoplasmic p53 and Foxo1 were significantly translocated to the nucleus in the cardiomyocytes of DOX-treated rats. Importantly, 6 weeks of oral CPT administration significantly suppressed p53 nuclear translocation and enhanced Foxo1 nuclear retention (Figure 7D).

## DISCUSSION

The present study confirmed that treating H9c2 cardiomyocytes with CPT at different time points did not lower cell viability. Further, 10μM CPT significantly suppressed DOX-induced reduction in H9c2 cardiomyocyte viability as detected using the CCK-8 assay. This pharmacological concentration was consistent with the results of previous studies [14, 18]. F-actin staining showed that CPT significantly increased the surface area of cardiomyocytes. DOX treatment alone significantly reduced the H9c2 cell surface area. Additionally, flow cytometry analysis showed that DOX significantly increased the ROS levels and reduced the MMP levels in cardiomyocytes compared with those in the control group. Importantly, CPT significantly suppressed the drastic ROS production and elevated MMP levels. It is generally believed that increased ROS production induced by oxidative stress due to cardiac mitochondrial deficiency is one of the widely accepted mechanistic basis in DOX-mediated cardiotoxicity [2, 12]. A previous study showed that enhancing the antioxidant capacity improved mitochondrial function but protected cardiomyocytes from damage caused by oxidative stress [12]. Moreover, CPT suppressed DOX-induced apoptosis of H9c2 cardiomyocytes *in vitro*.

If DOX-induced cardiotoxicity is not treated continuously, patients receiving doxorubicin treatment rapidly develop left ventricular dysfunction and cardiomyopathy, which eventually leads to heart failure [19, 20]. In the present study, rat cardiac function examined by echocardiography and hemodynamic monitoring showed that CPT significantly improved DOX-induced rat cardiac dysfunction. Next, cardiac histological changes were examined in Wistar rats treated with DOX. The histological changes caused by CPT treatment, including decreased cavitation in the nuclei and reduced fibrotic contents, were consistent with the recovery of rat cardiac insufficiency.

DOX-induced cardiac fibrosis is one of the most common characteristics of pathological cardiac remodeling [21]. ROS is believed to drive cardiac mesenchymal fibroblast activation and its trans-differentiation as well as promote the expression of extracellular matrix proteins relevant to increased collagen production [22]. Significant increases in the levels of ROS have been detected in various types of cardiac deficiency and/or heart failure [8]. Recent studies showed that removing damaged cardiomyocyte mitochondria could prevent the expression of apoptosis-related proteins and inhibit the apoptosis of cardiomyocytes [19]. *In vitro* experiments confirmed that CPT reduced the ROS levels and increased the MMP levels in H9c2 cells. The ROS levels in the heart tissue of the rats in the DOX + CPT group were significantly reduced. Additionally, oral CPT administration also increased the MMP levels in the cardiomyocytes of the left side of the heart of the rats. Both *in vivo* and *in vitro* experiments confirmed that CPT suppressed cardiomyocyte apoptosis through reducing intracellular ROS and increasing cardiac mitochondrial MMP levels. The present study indicated that CPT significantly improved the antioxidative capacity in the heart of DOX-treated rats and reduced the MDA levels.

Both KEGG and western blot analyses showed that the p53 signaling pathway was the key pathway involved in the CPT-mediated suppression of DOX cardiotoxicity. The present study found that CPT significantly decreased the expression level of 14-3-3σ in the heart of the DOX-induced cardiotoxicity rat model. 14-3-3σ is a member of the 14-3-3 family, and the members of this family are highly conserved acidic proteins widely expressed in tissue cells [23]. 14-3-3σ activation is dependent on AKT-ROS signaling stimulated by the diabetic pathophysiological factors [24]. Meanwhile, in an ischemia-perfusion injury model, increased JNK activation could promote mitochondria-mediated apoptosis through the mitochondrial translocation of pro-apoptotic proteins dependent on its release from 14-3-3σ [25]. Also, CPT treatment in rats injected with DOX leads to a significant reduction in 14-3-3σ levels accompanied by a reduction in JNK phosphorylation in the heart, which was consistent with the results of the aforementioned study. Like CPT, tanshinone IIA is also present in the extract of *danshen*. In the hypoxia-induced cardiomyocyte injury model, tanshinone IIA promotes 14-3-3η-mediated Bcl-2 translocation, which eventually protects mitochondrial function, prevents caspase-3 activation and inhibits apoptosis [26]. The present study showed that CPT significantly increased the Bcl-2 and Bcl-xl levels in the rat myocardium and suppressed caspase-9 and caspase-3 cleavage. Similarly, the expression levels of proteins related to mitochondria apoptosis including Bax, Bak and Bim, were also reduced by CPT. 14-3-3γ is another 14-3-3 family member involved in cardiac injury and protection [27, 28]. He et al. [29] showed that the upregulation of 14-3-3γ alleviated DOX-induced mouse cardiac injury, which was also via the suppression of DOX-induced oxidative stress and improvement in mitochondrial function. An earlier study found that 14-3-3σ regulated the p53 signaling pathway [30]. The results of the present study showed that CPT suppressed p53 nuclear translocation. p53 is a transcription factor for the expression of the *PUMA* gene, which is involved in regulating mitochondrial apoptosis [31, 32]. Similarly, CPT significantly downregulated the expression of PUMA protein. Additionally, AKT and the 14-3-3 family are involved in regulating the nuclear retention of the transcription factor Foxo1 [33, 34]. Consistent with these findings, the present study also found that CPT significantly enhanced AKT phosphorylation. The previous and present findings indicated that CPT enhanced Foxo1 nuclear translocation and retention through 14-3-3σ and AKT, which then suppressed the expression of proteins related to mitochondria apoptosis, such as Bim. Additionally, we believe that the present study still has some limitations. Some studies have found that cryptotanshione can inhibit the proliferation of tumor cells by enhancing ROS levels and inhibiting the activation of the MAPK-AKT signaling pathway [35, 36]. It is needed in the future to clarify how cryptotanshione regulates the MAPK-AKT signaling pathway and reactive oxygen levels in tumor and normal tissues, and what more novel mechanisms are at work.

**Figure 9:**
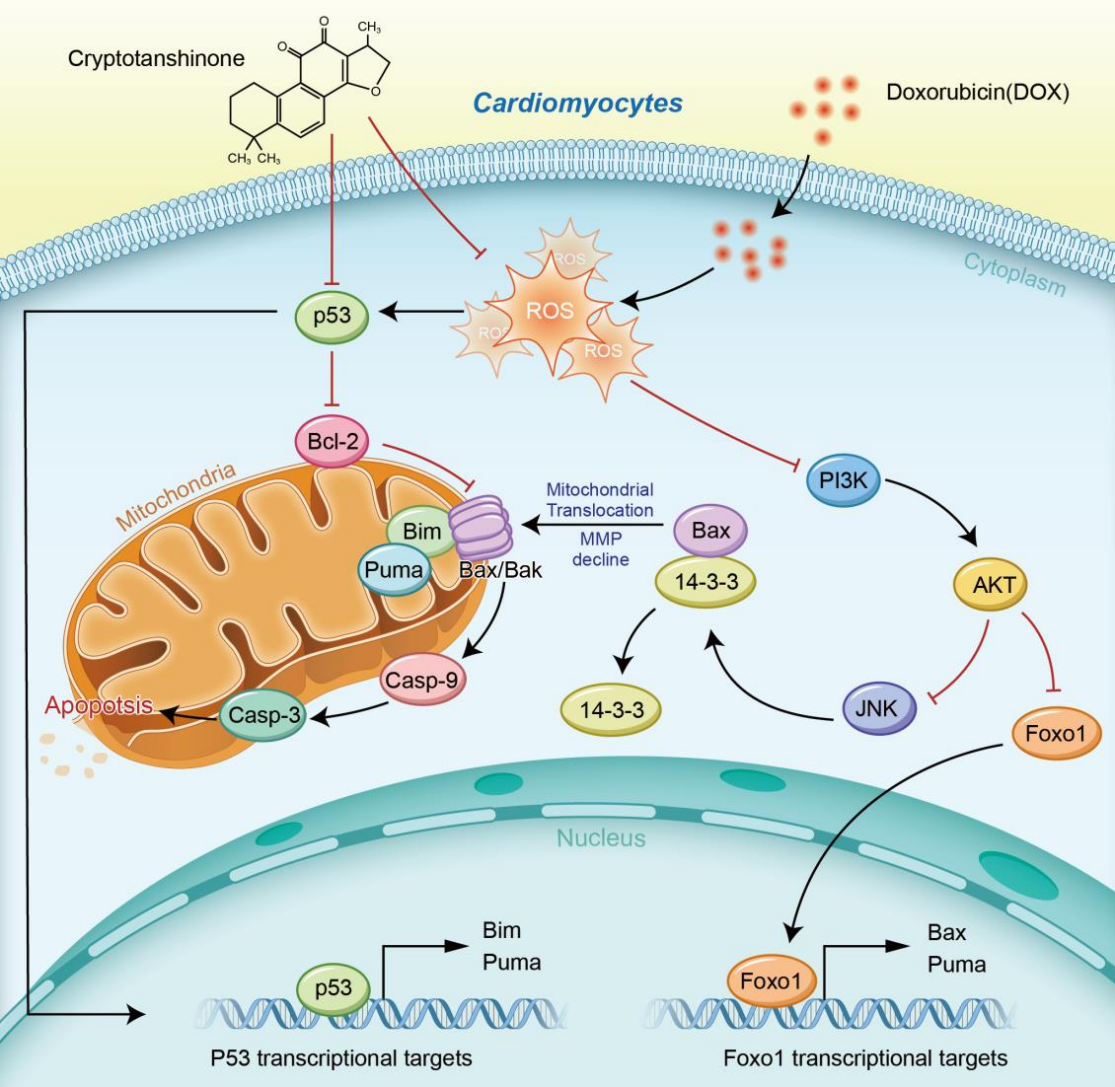
**Schematic diagram of the cardioprotective effects of cryptotanshinone (CPT) in a DOX-induced myocardial damage model.**

Taken together, *in vitro*, CPT significantly suppressed ROS production in cardiomyocytes, elevated MMP levels that were reduced by DOX, and alleviated DOX-induced cardiomyocyte apoptosis. It significantly improved DOX-induced cardiac dysfunction in rats, inhibited myocardial fibrosis and apoptosis, reduced DOX-induced ROS production in the heart, and elevated mitochondrial MMP levels in cardiomyocytes (Figure 9). Additionally, it enhanced the cardio-protective signaling activation in the p53 pathway and reduced the expression of proteins related to mitochondrial apoptosis. These observations suggested that CPT could be used as a novel anti-cardiotoxicity drug to prevent or delay the pathogenesis of doxorubicin-induced cardiac damages. Danshen has been used as a traditional medicinal plant for more than a thousand years in Northeast Asia. However, CPT, one of the extracts of danshen, still needs detailed analysis, including proteomics and metabolomics, and strict clinical trials for its application in the population treated with antineoplastic drugs.

## MATERIALS AND METHODS

### Drugs and chemical reagents

Doxorubicin hydrochloride for injection was produced by Haizheng-*Pfizer* Pharmaceutical Ltd. (Shanghai, China). The DOX used for *in vitro* experiments was a product of Solarbio (Beijing, China). CPT was purchased from the Desite Biotechnology Co., Ltd. (Chengdu, China) and MedChemExpress LLC (Shanghai, China).

### Cell culture and experimental design

The rat H9c2 cardiomyocyte cell line was acquired from the Cell Biology Laboratory of the Advanced Research Center in Central South University. Cardiomyocytes were cultured in the Roswell Park Memorial Institute (RPMI) 1640 medium (Gibco, Shanghai, China) containing 15% fetal bovine serum, 200 U/mL penicillin (Gibco) and 200 μg/mL streptomycin (Gibco) in an incubator supplemented with 5% CO_2_ at 37°C. The cells were plated in cell culture plates at a certain density and synchronized in serum-free RPMI 1640 medium for 6 h after culturing for 18 h. After synchronization, the RPMI 1640 complete culture medium containing 2, 5 and 10μM CPT was added to each well, with each concentration having three replicates. The H9c2 cell viability was assessed at 0, 3, 6 and 12 h after CPT treatment using the CCK-8 kit (Beyotime, Shanghai, China). Additionally, 1.0x10^4^ cells were plated onto a 96-well plate and divided into 5 groups: control group (Ct) treated with phosphate buffer saline (PBS), DOX (2μM) group, DOX (2μM) + CPT (2μM) group, DOX (2μM) + CPT (5μM) group, and DOX (2μM) + CPT (10μM) group. The cell viability was measured at 3, 6 and 12 h after the treatment, with each group having three replicates.

### F-actin staining and flow cytometry

H9c2 cardiomyocytes were plated onto a six-well glass-bottom cell culture plate (NEST, Wuxi, China). When the cells reached a confluence of approximately 60%, PBS, culture medium containing CPT (10μM) and DOX (2μM), and culture medium containing DOX (2μM) + CPT (10μM) were added and cultured for 12 h. The cells were then subjected to F-actin staining using phalloidin (Abcam, Shanghai, China). Images were taken from 10 random fields using a Zeiss fluorescence microscope, and the cell surface area was analyzed using Image-Pro Plus 6.2 software. The MMP level, ROS level and apoptosis of the rat H9c2 cardiomyocytes in each group were assessed using a JC-1 (5,5′,6,6′-Tetrachloro-1,1′,3,3′-tetraethyl-imidacarbocyanine iodide, JC-1) kit (Beyotime), a ROS detection kit (DOJINDO, Shanghai, China) and an Annexin V-FITC (fluorescein isothiocyanate, FITC) apoptosis detection kit (BD Bioscience, CA, USA) respectively, according to the manufacturers’ protocols.

### Animal model and experimental design

The animals used in this study were male Wistar rats, weighing 220−260 g, acquired from the SJA Laboratory Animal Co., Ltd. (Hunan, China). Housing and experimental procedures were performed in accordance with the guidelines of the Experimental Animal Center of Hunan Province, Department of Laboratory Animal Sciences of Xiangya School of Medicine, Central South University and Hunan Provincial Laboratory Animal Public Service Center. All rats were housed in a specific pathogen free (SPF) environment under a light−dark cycle with free access to food and water.

A total of 28 Wistar rats were randomly divided into the healthy control group (Ct), healthy control with oral administration of CPT group (Ct + CPT), DOX treatment group (DOX), and DOX + CPT treatment group (DOX + CPT), with 7 rats in each group. The detailed treatments were as follows:

1. The rats in the Ct group (*n=7*) were orally treated with 1 mL of saline (0.9% sodium chloride solution) every day.
2. The rats in the Ct + CPT group (*n=7*) were orally administered with CPT at a dose of 50mg/kg once every day for a total of 6 weeks.
3. The rats in the DOX group (*n=7*) were injected with doxorubicin hydrochloride at a dose of 2 mg/kg, with three injections each week and a total of six injections.
4. The rats in the DOX + CPT group (*n=7*) were injected with doxorubicin hydrochloride at a dose of 2 mg/kg, with three injections each week and a total of six injections. Meanwhile, the rats in this group were orally administered with CPT at a dose of 50 mg/kg [14] once every 2 days for a total of 6 weeks.

After intervention, some rats were randomly selected from each group and subjected to echocardiography and hemodynamic examination. Then, all the rats were sacrificed according to the animal welfare regulations. Blood was extracted and treated with heparin for anticoagulation. Plasma was isolated after centrifugation and stored in a −80°C freezer for future use. The heart was isolated, and parts of the left ventricle were stored in liquid nitrogen or soaked in TRIzol reagent (Thermo Fisher Scientific, Shanghai, China) for the subsequent experiments. The remaining tissue was soaked in 10% paraformaldehyde and stored at room temperature.

### Evaluation of cardiac function

At the end of the experiment, three rats were randomly selected from each group for echocardiography. First, the rats were anesthetized with 20% urethane solution. The cardiac function was then examined using the Vivid 7.0 Doppler ultrasound (GE, Illinois, USA); an ultrahigh-frequency probe was used (10 MHz). The LV function was assessed from the parasternal long- and short-axis views at the apical level of the mitral valve. End systole and diastole volumes were defined as the LV minimum and maximum capacities, respectively. The EF% and FS% were measured using the LV-M mode with a scanning speed of 50 mm/s.

Hemodynamic examination is an invasive means of assessing the changes in the LV pressure and volume in the animal being assessed. At the end of the observation, each rat was anesthetized with a 20% urethane solution at a dose of 5 mL/kg. A catheter (PowerLab, ADInstrument, New Zealand) with a pressure-responsive electrode (Millar, Texas, USA) was inserted at the proximal end through the left carotid artery of the rat. After inserting the head of the catheter with the pressure-responsive electrode into the LV of the rat, the PV-loop data was recorded when a smooth and stable pressure loop appeared.

### Determination of oxidative and antioxidative stress markers

The supernatants of rat cardiac tissue homogenates were used to measure the activities of antioxidative stress-related enzymes and non-enzymatic substances. SOD, GSH-Px, CAT, and the nonenzymatic substance glutathione were measured using commercially available kits from Beyotime Biotechnology Inc. (Shanghai, China) and the Jiancheng Bioengineering Institute (Nanjing, China). The lipid peroxidation product MDA was measured using a kit from Solarbio Life Sciences (Beijing, China).

### Histochemistry staining analysis

The rat heart fixed in paraformaldehyde was embedded in paraffin and sectioned at a thickness of 4−6 μm. The tissue sections were subjected to HE staining and MTS, as previously described. Images were taken by two independent investigators using inverted microscopy and analyzed using Image-Pro Plus 6.0.

### Measurements of MMP and ROS

MMP was measured using the mitochondrial membrane potential assay kit with a JC-1 kit (Beyotime Biotechnology). Conversion of JC-1 from red fluorescence into green fluorescence is an early marker for apoptosis. The probe DCFH-DA (2',7'-Dichlorodihydrofluorescein diacetate) (Beyotime Biotechnology) was used to determine the ROS levels in the frozen cardiac tissue sections. Intracellular ROS oxidizes nonfluorescent DCFH to form fluorescent DCF. The fluorescent signals of DCF were evaluated for intracellular ROS levels.

### TUNEL assay

Apoptosis was assessed by TUNEL. Cardiomyocyte apoptosis in the 4-μm-thick frozen sections was examined using the TUNEL one-step fluorescence staining kit (Beyotime Biotechnology) according to the manufacturer’s protocol. Apoptotic cells were shown in green, and nucleus 4',6-diamidino-2-phenylindole (DAPI) staining was shown in blue. Some cardiomyocytes from eight randomly selected fields were counted from each animal heart tissue sections and the apoptosis rate were calculated using number of TUNEL-positive cells/number of total cells.

### Transcriptomic profiling and KEGG analysis

The total RNA from the cardiac tissue was extracted using the Trizol reagent (Thermo Fisher Scientific, Shanghai, China) according to the manufacturer’s protocol. The total RNA was extracted from the sample. After DNA digestion using DNase, mRNA was enriched with oligo (dT)–coupled magnetic beads. The mRNA was broken into short fragments using the breaking reagent. Single-stranded cRNA was synthesized with the six-base random primers using the broken mRNA as templates. Double-stranded cDNA was then synthesized in the double-stranded reaction system and purified. Purified double-stranded cDNA was end-repaired, tagged with an A tail, ligated to the sequencing linker, subjected to fragment size selection and then subjected to PCR amplification. After the qualification analysis using the Agilent 2100 Bioanalyzer, the constructed cDNA library was sequenced using the Illumina HiSeq X Ten sequencer to generate double-ended data of 125 or 150 bp. The raw imaging data files from the high-throughput sequencing were converted into the original sequencing results by base calling, which were called raw data or raw reads. The results were stored in the FASTQ file format, which included the sequencing information of the reads and its corresponding sequencing quality information. A large number of sample double-ended sequencing data were acquired through the Illumina platform. Due to the impact of the data error rate on the results, Trimmonatic [37] was used to perform quality preprocessing on the raw data and statistically summarize the number of reads in the entire quality control process. HISAT2 [20] was used to compare the sequence of clean reads with the specified reference genome and acquired their locations on the reference genome or genes as well as the sequence characteristic information specific to the sequenced sample. The expression level of protein-coding genes was calculated by the fragments per kb per million reads (FPKM) method [38], which showed the number of fragments per kilobase length from a protein-encoding gene per million fragments. FPKM considered the effects of both the sequencing depth and the length of protein-coding genes on the counting of fragments. It is currently the most common method for evaluating the expression levels of protein-encoding genes. Differential expression analysis aimed to identify differentially expressed genes among different samples. After acquiring the differentially expressed genes, the gene ontology (GO) functional significance and KEGG pathway significance analyses were performed [39]. The transcriptomic profiling and bioinformatics analysis were conducted by OE Biotech Co., Ltd. (Shanghai, China).

### Western blot analysis

The cardiac tissue from LV (approximately 20 mg) was homogenized in a homogenizer and lysed in radio immunoprecipitation assay (RIPA) buffer (Abcam, Shanghai, China) containing protease and phosphatase inhibitors (MedChemExpress LLC) for 5−10 min. The lysates were centrifuged at 12,000*g* for 10 min at 4°C, and the supernatants were collected for cardiac protein analysis. The protein concentration was measured using the bicinchoninic acid (BCA) protein assay kit (BosterBio, Wuhan, China) according to the manufacturer’s protocols. Denatured proteins were separated by 6−12% sodium dodecyl sulfate- polyacrylamide gel electrophoresis (SDS-PAGE) and transferred onto the polyvinylidene fluoride (PVDF) membrane (Amersham, GE Healthcare, Germany). The membrane was blocked in a 5% bovine serum albumin (BSA) solution (BosterBio) for 2 h at room temperature and incubated with the primary antibody overnight at 4°C. The primary antibodies used in this study included 14-3-3σ (mouse, 1:1000 dilution; Santa Cruz Biotechnology, Shanghai, China), PI3Kinase p85 (rabbit, 1:1000 dilution; Cell Signaling Technology, Inc., Shanghai, China), p-AKT (rabbit, 1:1000 dilution; Cell Signaling Technology, Inc.), AKT (rabbit, 1:1000 dilution; Cell Signaling Technology, Inc.), p-JNK (rabbit, 1:1000 dilution; Cell Signaling Technology, Inc.), JNK (rabbit, 1:1000 dilution; Cell Signaling Technology, Inc.), Bcl-2 (mouse, 1:1000 dilution; Santa Cruz Biotechnology), Bcl-xl (rabbit, 1:1000 dilution; Cell Signaling Technology, Inc.), Bax (rabbit, 1:1000 dilution; Cell Signaling Technology, Inc.), Bak (rabbit, 1:1000 dilution; Cell Signaling Technology, Inc.), Bim (rabbit, 1:1000 dilution; Cell Signaling Technology, Inc.), Puma (rabbit, 1:1000 dilution; Cell Signaling Technology, Inc.), p53 (mouse, 1:1000 dilution; Cell Signaling Technology, Inc.), Foxo1 (rabbit, 1:1000 dilution; Cell Signaling Technology, Inc.), caspase-9 (mouse, 1:1000 dilution; Santa Cruz Biotechnology), caspase-3 (rabbit, 1:1000 dilution; Cell Signaling Technology, Inc.), GAPDH (mouse, 1:1000 dilution; OriGene, Beijing, China), PCNA (mouse, 1:1000 dilution; OriGene) and β-tubulin (mouse, 1:1000 dilution; OriGene). The secondary antibodies used in this study were goat anti-mouse IgG (peroxidase conjugated, H+L; Millipore, MA, USA) diluted at 1:5000 and goat anti-rabbit IgG (peroxidase conjugated, H+L; BosterBio) diluted at 1:1000. The membrane was incubated with secondary antibodies for 2 h. The membrane was exposed using the chemiluminescent HRP substrate (Millipore), and the luminescence of the membrane was detected using a chemiluminescent imager (SmarChemi^TM^, SAGECREAATION, Beijing, China).

### Statistical analysis

SPSS 16.0 was used for statistical analysis. Data were subjected to one-way analysis of variance, chi-squared test and *t*-test. Quantitative data were represented by mean ± standard deviation. A *P* value less than 0.05 was considered statistically significant.
